# Study on the quality characteristics of jujube slices under different pretreatment and drying methods

**DOI:** 10.1016/j.ultsonch.2025.107305

**Published:** 2025-03-06

**Authors:** Zhengdong Wan, Zhuofan Ji, Dandan Zhao, Yamei Liu, Zhentao Zhang, Jianxiong Hao

**Affiliations:** a College of Food Science & Biology, Hebei University of Science & Technology, Shijiazhuang 050018, China; b Hebei Provincial Functional Food Technology Innovation Center, Shijiazhuang 050018, China; c Technical Institute of Physics and Chemistry CAS, Beijing 100190, China

**Keywords:** Jujube slices, Pretreatment methods, Drying techniques

## Abstract

This study investigates the effects of different pretreatment methods, cold plasma (CP) and ultrasound (US), as well as different drying techniques, including vacuum freeze-drying (FD), hot air drying (HAD), and microwave coupled with pulsed vacuum drying (MPVD), on the quality characteristics of winter jujube slices. The physical, chemical, and functional properties were analyzed, encompassing farinograph attributes, particle size, cation exchange capacity, total phenolic and flavonoid content, and flavor compounds were analyzed. In terms of physical properties, jujube slices subjected to MPVD demonstrate superior water-holding capacity at 2.93 g/g and enhanced fluidity, with a sliding angle of 34.98° and an angle of repose of 43.47°, compared to FD jujube slices. Additionally, it exhibits a rehydration capacity of 2.98 g/g and a bulk density of 0.49 g/mL. Regarding chemical composition, the cation exchange capacity of US-FD jujube slices is measured at 0.64 mmol/g, while the total phenolic content reaches 11.97 mg/g, and the flavonoid content in CP-MPVD jujube slices is 5.21 mg/g. Notably, the cation exchange capacity of MPVD jujube slices pretreated by CP and US is 0.46 and 0.55 mmol/g, respectively. Concerning volatile compounds and flavor, FD slices retain higher concentrations of aldehydes (11.39 %) and alkenes (16.88 %), whereas MPVD slices contain 21.20 % alkanes. HAD slices contain the highest aromatic hydrocarbon content at 34.97 %. In summary, the optimized combination of CP and MPVD can enhance the drying efficiency of jujube slices, increase the flavonoid content in jujube slices, and improve their quality characteristics.

## 1 Introduction

The jujube fruit, taxonomically classified as *Zizyphus* within the Rhamnaceae family, has a cultivation history spanning more than 4000 years and is widely dispersed across northern China [1]. Jujube fruit is rich in diverse bioactive constituents, namely phenols, flavonoids, polysaccharides, organic acids, and anthocyanins [2]. Modern pharmacological studies have demonstrated that jujube fruit possesses a wide range of beneficial health-promoting properties, including sedative and hepatoprotective effects, immunological and antioxidant activities, as well as anti-inflammatory effects [3].

Fresh jujubes have a high-water content and a short storage period. If stored improperly, they will easily deteriorate. And jujube powder has recently been proposed as the best product to use in many food formulations to develop functional foods [4]. The jujube powder can be used to produce various products such as jujube wine and solid beverages, presenting a broad market prospect. Nevertheless, there is still limited development and utilization of jujube powder in the market, making it particularly important to develop jujube powder with high bioactive components and rich nutrients. In the food industry production, processing fresh jujubes into jujube powder helps maintain and improve nutritional content and flavor. For instance, the physical characteristics of jujube powder are primarily represented by its color and particle size. During food processing, its color contributes a natural hue to the product, thereby impacting its overall appearance. An appropriate particle size is crucial for ensuring the even distribution of jujube powder, which helps to avoid local taste discrepancies that may affect the product’s overall texture [5]. Cao et al. [6] emphasized that the real − time monitoring system they developed can effectively monitor the color and moisture content changes of jujube slices during the drying process, predict the vitamin C and reducing sugar content, and provide a useful reference for real − time quality monitoring of fruits and vegetables during drying by means of machine vision, automatic weighing, and the establishment of correlation models. Concurrently, jujube fruits are rich in polyphenolic flavonoids and various vitamins, which has significantly increased people’s interest in the research and development of jujube fruit products [7]. Consequently, advancements in pretreatment technology and drying methods are pursued to achieve a more refined product structure and enhanced nutrient content.

Drying is a critical process in food preservation that significantly impacts the quality and shelf-life of products. Various drying methods have been developed to remove moisture from fruits, including hot air drying (HAD), freeze drying (FD), and microwave pulsation vacuum drying (MPVD), each with its unique advantages and limitations. HAD is the most traditional method, which involves circulating hot air around the product, effectively reducing moisture content. This method is popular due to its simplicity and low cost, making it suitable for large-scale production but may lead to nutrient loss [8]. FD preserves the structural integrity, flavor, and nutritional content of the fruit by freezing it and then removing moisture through sublimation. However, a significant drawback of FD is its lengthy duration and high energy consumption, which hinders the application of this technology in large-scale industrial production [9]. MPVD is an advanced drying technology that combines the advantages of microwave heating and vacuum environment. In a vacuum environment, the boiling point of water is lowered, making it easier to vaporize and penetrate directly into the material, causing the inside and outside of the material to be heated at the same time [10]. Due to the rapid drying process, there is less damage to some heat-sensitive substances such as vitamins and biologically active ingredients [11]. And our research group has also published research articles on the quality analysis and pectin characteristics of MPVD of winter jujubes [12]. In the pulsating vacuum environment, the material is in relatively mild drying conditions, reducing nutrient loss caused by long-term heating or oxidation. However, these new technologies still face some challenges in practical applications, such as high equipment costs and complex operations. Therefore, it is of great practical significance to explore more efficient and low-cost drying pretreatment methods that can ensure product quality.

Pretreatment is an essential step in the processing of jujube powder that can significantly enhance the quality and functionality of the final product. Various pretreatment methods, such as ultrasound (US), cold plasma (CP), and high-pressure processing (HPP), are employed to prepare the jujube for drying [13]. Yuan et al. [14] reported that CP and HPP pretreatments improve the quality characteristics of vacuum freeze-dried jujube slices by preserving color, texture, and bioactive compounds, making them suitable for high-quality processing. Similarly, da Silva et al. [15] highlighted that ultrasonic-assisted freeze-drying is an efficient method for enhancing the quality attributes of fruits and vegetables by improving mass transfer during drying. Mirzaei-Baktash et al. [16] reported that the drying time of mushroom pretreated by ultrasonic treatment was obviously shortened. Jiang et al. [17] emphasized that freeze-ultrasound (FU) pretreatment is an effective means to improve the infrared combined hot air impingement drying efficiency of strawberry slices and improve their cell ultrastructure, enzyme activity and physicochemical properties by enhancing cell membrane permeability. Ni et al. [18] emphasized that surface discharge cold plasma treatment can efficiently degrade imidacloprid in water, clarify its degradation pathway, and reduce its residual toxicity by means of the effective participation of reactive oxygen species in the decomposition reaction. CP, a relatively new technology, can effectively inactivate microorganisms while preserving the fruit’s nutritional content and sensory attributes. Wang et al. [19] demonstrated that dehydration methods, including pretreatments, significantly influence the antioxidant activities and phenolic content of jujube fruits, emphasizing the role of pretreatment in retaining valuable bioactive compounds like cyclic nucleotides and volatiles. This method enhances the antibacterial properties of jujube, making it more shelf stable. High-pressure processing alters the cellular structure of jujube, increasing permeability and promoting moisture removal during subsequent drying. Furthermore, Zhang et al. [20] reported that active components and antioxidant activity in different varieties of jujube fruits are highly influenced by processing conditions, reinforcing the importance of optimizing pretreatment methods. And our research group has also published on the impact of pretreatment processing on drying characteristics [21]. By employing these pretreatment methods, producers can not only improve drying efficiency but also enhance the bioactive compounds in jujube powder, such as phenols and flavonoids, thereby increasing its nutritional value and potential health benefits. Overall, effective pretreatment strategies are vital for optimizing jujube slices processing and maximizing its utility in various food applications.

This study endeavored to 1) explore the impacts of diverse pretreatment and drying approaches on multiple aspects of winter jujube slices, including its color, physical features, cation exchange capacity, total phenolic content, flavonoid active constituents, and flavor substances; 2) screen and appraise the most favorable processing method for jujube, thereby laying a theoretical groundwork for its processing. It is expected to alleviate the wastage of fresh winter jujube caused by its short storage life and augment the economic worth of winter jujube-derived products.

## 2 Materials and methods

### 2.1 Raw materials

The raw materials were Huanghua winter jujubes purchased from Cangzhou City, Hebei Province, China. The maturity stages of winter jujubes are divided into early maturity and natural maturity. The natural maturity period is from October to November, and the early maturity period is from August to September. For this research, winter jujubes that reach natural maturity in October exhibit no surface damage and are consistent in size have been meticulously selected. They are transported to the laboratory after 2–3 days and stored in a refrigerator at 4 ± 1 °C. The initial moisture content of the material is 3.9 ± 0.6 (g water/g dry basis), and the average sugar content is 31.5 %.

### 2.2 Drying experiment and pretreatment experiment

Before the drying experiment and pretreatment experiment, fresh jujube fruits were cored and sliced into uniform pieces measuring 5 mm.

#### 2.2.1 Freeze drying (FD)

About 100.0 ± 1.0 g of fresh jujube slices were placed in a ziplock bag and stored at −80 °C for 24 h for pre-freeze. Subsequently, the pre-frozen samples were taken out and transferred to a vacuum freeze dryer (FD1600-A, Qingdao Fullum Technology Co., Ltd., China). The cold trap temperature of the vacuum freeze dryer was maintained at −40 °C with a pressure of 40 Pa. The samples were left in the dryer for 48 h before taken out. All the drying process was repeated three times.

#### 2.2.2 Microwave coupled with pulsed vacuum drying (MPVD)

About 50.0 ± 1.0 g of fresh jujube slices was dried by microwave vacuum dryer (ORW1.0S-8Z, Nanjing Aorun Microwave Technology Co., Ltd., China) with a microwave power of 250 W (5 W/g) and a vacuum degree of −90 Kpa. All the drying process was repeated three times.

#### 2.2.3 Hot air drying (HAD)

About 100.0 ± 1.0 g of fresh jujube slice was dried by hot air convection dryer (DHG-9140A, Shanghai Jinghong Laboratory Equipment Co., Ltd., China) at 60 °C with the air velocity of 1 m/s. The weight of samples was recorded every 30 min until the moisture content on wet was 10 %. All the drying process was repeated three times.

#### 2.2.4 Cold plasma (CP)

Fresh winter jujube slices should be placed between the electrodes (CTP-2000 K, Nanjing Suman Plasma Technology Co., Ltd., China), ensuring that the surface of the material is positioned 5 mm from the electrodes. The voltage and current values should be set to 50 V and 2 A, respectively, with a processing time maintained at 30 s. Following this, the samples were subjected to three distinct drying methods: FD, MPVD, and HAD. Three experiments were conducted for each drying method.

#### 2.2.5 Ultrasound (US)

Fresh winter jujube slices should be immersed in 500 mL of water and subsequently subjected to ultrasonic pretreatment using an experimental ultrasonic cleaning machine (KQ-250DE, Kunshan Ultrasonic Instruments Co., Ltd., China). The ultrasonic power is set to 200 W, with a frequency of 40 kHz, a duration of 20 min, and a temperature maintained at 25 °C. Following this pretreatment, the samples were subjected to three distinct drying methods: FD, MPVD and HAD, with three experimental replications conducted for each drying method.

#### 2.2.6 Control

Fresh winter jujubes were dried using a microwave pulse vacuum dryer at 250 W (5 W/g), with a processing batch of 50 g per run. Hot air drying at 60℃ and vacuum freeze-drying were used as controls. After drying, the jujubes were processed into powder using a universal grinder (FSJ302-5, Tianjin Test Instrument Co., Ltd., China) with each grinding cycle lasting 10 s, followed by a 5-minute interval, for a total of three cycles. The powder was then passed through a 40-mesh sieve and further ground using a disc-type ultrafine grinder to obtain a fine powder.

### 2.3 Quality characteristics of apricot slices

#### 2.3.1 Color measurement

Jujube powder was evenly spread on a glass dish, and a colorimeter (CR-400, Konica Minolta., Japan) was employed to measure the color difference of the powder. The color was characterized using L*, a*, and b* values, and the color difference (ΔE) of the samples was subsequently calculated. Each sample was measured six times, and the average value was recorded. The calculation of the color difference was based on Equation (1) [22]:

(1) $$\Delta E=\sqrt{{\left(L_{0}^{*}-L^{*}\right)}^{2}+{\left(a_{0}^{*}-a^{*}\right)}^{2}+{\left(b_{0}^{*}-b^{*}\right)}^{2}}$$

Where:

*ΔE* represents the color difference of the dried jujube samples,

L*, a*, and b* are the color values of the dried jujube samples,

$L_{0}^{*}$, $a_{0}^{*}$, and $b_{0}^{*}$ are the color values of the vacuum freeze-dried samples.

#### 2.3.2 Particle size distribution

A particle size analyzer (BT-9300S, Dandong Bettersize Instruments Ltd., China) was used to measure the particle size distribution of the dried jujube powder. The particle size of the powder was characterized by *D*_0.1_, *D*_0.5_, *D*_0.9_, and span. *D*_0.1_, *D*_0.5_, *D*_0.9_ represent the volume diameters corresponding to the cumulative particle size distribution of 10 %, 50 %, and 90 % of the samples, respectively. The span calculation was based on Equation (2):

(2) $$\mathrm{Span}=\frac{D_{0.9}/\mu m-D_{0.1}/\mu m}{D_{0.5}/\mu m}$$

#### 2.3.3 Bulk density measurement

Two grams of jujube powder were weighed and placed into a 10 mL graduated cylinder. The samples were oscillated with a vortex shaker for 1 min to stabilize the volume. The volume of the samples was recorded, and the bulk density was calculated as the ratio of the mass of the jujube powder to the volume of the cylinder. Each sample was measured three times, and the average value was taken.

#### 2.3.4 Rehydration ratio measurement (RR)

The RR was determined followed by Wang et al. [23]. Jujube powder (1 g) was placed into a 50 mL centrifuge tube, followed by the addition of 20 mL of distilled water. The samples were left to stand for 1 h at 25℃. It was then centrifuged (HC-3018, Anhui Zhongke Zhongjia Scientific Instruments Co., Ltd., China) at 10,000 r/min for 25 min, and the supernatant was removed. The mass of the sediment was recorded. The measurement was repeated three times, and the average value was taken. The rehydration ratio was calculated according to Equation (3):

(3) $$\mathrm{RehydrationRatio}=\frac{m_{2}-m_{1}}{m_{1}}$$

where *m*_1_ and *m*_2_ represent the mass of the jujube powder before and after rehydration, respectively, g.

#### 2.3.5 Measurement of repose angle and sliding angle

The measurement of the repose angle and sliding angle was conducted according to the method reported by Sun et al. [24] in their study on repose angle and critical sliding angle of snow on curved roofs.

For the repose angle measurement, coordinate paper was placed flat on the tabletop. Two funnels were prepared and fixed so that the bottom of the upper funnel was aligned with the inner wall of the lower funnel. The lower funnel was positioned 6 cm (H) above the coordinate paper. The samples powder was poured continuously into the upper funnel, allowing it to flow freely until the apex of the cone formed by the powder touched the outlet of the funnel. The radius of the cone on the coordinate paper (R/cm) was recorded. This process was repeated three times, and the average value was taken. The repose angle was calculated using Equation (4):

(4) $$\text{Re}pose\;Angle=\arctan\left(\frac{H}{R}\right)$$

For the sliding angle measurement, a glass plate measuring 10 cm in length (H) and 6 cm in width was placed horizontally on the tabletop with one end fixed at the base. Precisely 1 g of jujube powder was placed on the free end of the glass plate. The free end was then slowly raised until the jujube powder began to slide. The height of the raised glass plate (L/cm) now of sliding was recorded. This process was repeated three times, and the average value was taken. The sliding angle was calculated according to Equation (5):

(5) $$\mathrm{Sliding}\;Angle=\arcsin\left(\frac{L}{H}\right)$$

#### 2.3.6 Measurement of water-holding capacity and solubility

The measurement of water-holding capacity and solubility was conducted following the method reported by Corrêa et al. [25] with slight modifications. Their study investigated the dehydration and rehydration behavior of osmotic pretreated paneer slices (Indian cottage cheese) and employed a modeling approach.

For the water-holding capacity measurement, the mass of the centrifuge tube was first recorded as *m*. Then, 0.05 g of jujube powder was weighed and recorded as *m*_1_ and added to the centrifuge tube. Subsequently, 10 mL of distilled water gradually added while shaking the tube to ensure the powder was fully dissolved. The tube was then placed in a 60 °C water bath for 30 min, followed by cooling in cold water for 30 min. After cooling, the tube was centrifuged at 10,000 r/min for 40 min. Upon completion of centrifugation, the supernatant was collected, and the mass of the centrifuge tube with the sediment was recorded as *m*_3_. The water-holding capacity was calculated using Equation (6):

(6) $$\mathrm{Water}-HoldingCapacity\left(g/g\right)=\frac{m_{3}-m}{m_{1}}$$

For the solubility measurement, the supernatant from the centrifugation was transferred to an aluminum dish with an initial mass of *m*_2_. The aluminum dish with the supernatant was then dried at 105 °C to constant weight, and the mass of the aluminum dish with the remaining residue was recorded as *m*_3_. This process was repeated three times, and the average value was taken. The solubility index was calculated according to Equation (7):

(7) $$\mathrm{So}\mathrm{lub}ility\;Index=\left(1-\frac{m_{3}-m_{2}}{m_{1}}\right)\times 100\%$$

#### 2.3.7 Measurement of hygroscopicity

To measure hygroscopicity, 1 g of jujube powder was placed in a pre-weighed, dry aluminum dish. The aluminum dish was then placed inside a glass desiccator containing a saturated sodium chloride solution, maintaining a relative humidity of 75.5 %. The samples were stored for 7 days. Hygroscopicity was expressed as the mass of water absorbed per 100 g of dry matter. The hygroscopicity was calculated using Equation (8):

(8) $$\mathrm{Hygroscopicity}=\frac{m_{1}-m_{0}}{m_{0}}\times 100\%$$

where:

*m*_0_ is the initial mass of the jujube powder, g,

*m*_1_ is the mass of the jujube powder after moisture absorption, g.

#### 2.3.8 Measurement of microstructure

The morphology of the jujube powder was observed using Scanning Electron Microscopy. A small amount of the samples was evenly spread onto an adhesive pad, followed by gold sputter-coating. Observation was conducted at an accelerating voltage of 2.0 kV and a magnification of 500x.

#### 2.3.9 Measurement of cation exchange capacity

The cation exchange capacity (CEC) was measured following the method described by Younis et al. [26] in their study on the effects of thermal and pulsed electric field processing on the physicochemical properties of milk-based jujube powder beverages. A 0.5 g samples was weighed and mixed with 10 mL of 0.1 mol/L hydrochloric acid solution. The mixture was left at room temperature for 24 h. Afterward, the samples were filtered through filter paper, and the residue was thoroughly washed with distilled water to remove any residual hydrochloric acid.

The residue was then transferred to an Erlenmeyer flask, and 100 mL of a 15 % sodium chloride solution was added. The solution was agitated using a magnetic stirrer for half an hour. A 0.5 % phenolphthalein solution served as the indicator, while the mixture underwent titration with a 0.1 mol/L sodium hydroxide solution. Additionally, a blank test was performed by substituting hydrochloric acid with distilled water to measure the volume of sodium hydroxide used in the blank test. Each samples measurement included a blank control.

The cation exchange capacity was calculated using Equation (9):

(9) $$CEC\left(\mathrm{mmol}/g\right)=\frac{\left(V_{0}-V_{1}\right)\times 0.1}{m}$$

where:

*V*_0_ is the volume of sodium hydroxide consumed by the samples, mL,

*V*_1_ is the volume of sodium hydroxide consumed in the blank, mL,

*m* is the mass of the samples, g,

0.1 is the concentration of the sodium hydroxide solution, mol/L

#### 2.3.10 Determination of total phenolic content

Extraction of Total Phenolics: A total of 1 g of dried samples was mixed with 40 mL of an 80 % methanol solution. This combination underwent ultrasonic treatment in an ultrasonic cleaner for 30 min at ambient temperature. Following sonication, the mixture was centrifuged at 10,000 rpm for a duration of 10 min using a refrigerated centrifuge. The supernatant was collected, and the remaining residue was re-extracted on two additional occasions. All collected supernatants were then combined and adjusted to a final volume of 100 mL in a volumetric flask. The extract was preserved at 4 °C and should be utilized within 24 h.

Determination of Total Phenolics: The colorimetric Folin-Ciocalteu method was used. In a test tube, 0.8 mL of the extract was mixed with 4 mL of 10 % Folin reagent and 6 mL of 10 % sodium carbonate solution. The mixture was allowed to react in the dark for 2 h, after which the absorbance was measured at 765 nm. Gallic acid was used as the standard, and the standard curve was represented by the equation *y* = 0.005*x*−0.0062 (*R*^2^ = 0.9988). The total phenolic content of the dried jujube samples was calculated based on this curve and expressed in milligrams of gallic acid equivalents per gram of dry weight (mg GAE/g DW).

#### 2.3.11 Determination of total flavonoid content

Extraction of Total Flavonoids: The extraction procedure was the same as that used for total phenolics.

Determination of Total Flavonoids: The content of total flavonoids was determined using the NaNO_2_-Al(NO_3_)_3_-NaOH method. In a test tube, 0.4 mL of a 5 % sodium nitrite solution was combined with 4 mL of the extract. After a period of 6 min, 10 mL of a 10 % aluminum nitrate solution was introduced and permitted to react for an additional 6 min. Following this, 4 mL of a 4 % sodium hydroxide solution was added, and the mixture was allowed to react for 15 min. The mixture was then diluted to a final volume of 10 mL. Using methanol as a blank control, the absorbance was measured at 510 nm. Rutin was used as the standard, and the standard curve was represented by the equation *y* = 0.0034*x* + 0.0013 (*R*^2^ = 0.9985). The flavonoid content in the jujube samples was calculated based on this curve and expressed as milligrams of rutin equivalents per gram of dry weight (mg RE/g DW).

#### 2.3.12 Determination of flavor compounds

The measurement of volatile flavor compounds in the winter jujube samples was carried out following the methodology described by Benahmed eta al. [27]. Using gas chromatography-mass spectrometry (GC–MS), the composition and concentration differences of volatile compounds in jujube powder were analyzed across different pretreatment and drying methods. A 1 g samples was weighed and sealed in a 15 mL vial, then equilibrated at 50 °C for 40 min with an extraction time of 30 min. The extraction solution was introduced into the GC–MS injection port, with a desorption temperature of 250 °C for 5 min. Qualitative analysis of the aroma compounds in the jujube powder was conducted using mass spectrometry (MS) and retention index (RI) methods.

For the GC–MS system, a DB-5 capillary column (60 m × 0.25 mm × 0.25 µm) was utilized, with helium as the carrier gas at a flow rate of 1 mL/min. The mass spectrometry conditions included an electron impact (EI) ionization source with electron energy set at 70 eV. The mass spectrometer interface temperature was set to 250 °C, and the ion source temperature was maintained at 230 °C. Mass spectra were scanned over a range of 30–350 *m*/*z*. The percentage content of flavor compounds in the samples was calculated using the following formula (10):

(10) $$C_{x}=\frac{A_{X}}{A_{t}}\times 100\%$$

where *C_x_* is the percentage content of the flavor compound in the samples, *A_x_* is the peak area of each component, and *A_t_* is the total peak area.

#### 2.3.13 Data processing

All data were obtained as the mean values of results from at least three independent trials and are expressed as mean ± standard deviation. Statistical analysis was performed using SPSS Statistics 19.0 software, applying Duncan’s multiple range test for significance analysis at a significance level of P < 0.05. All graphs and charts were created using Origin 2021 software.

## 3 Results and analysis

### 3.1 Color

**Fig. 1:**
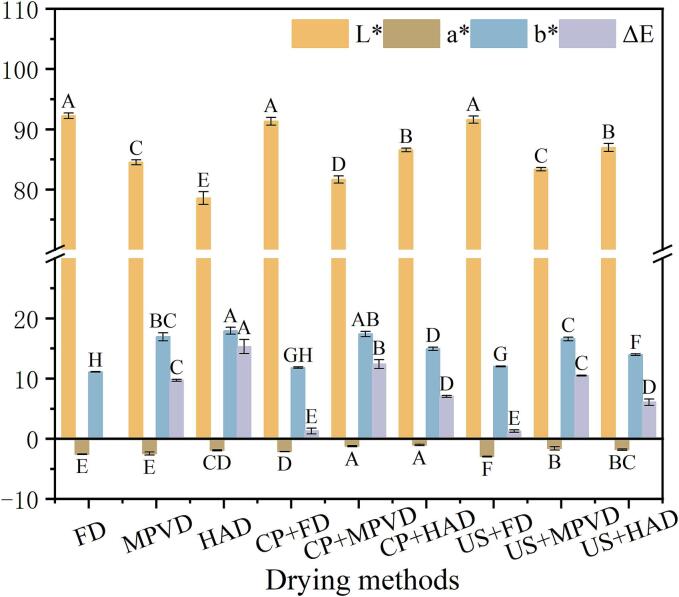
Effects of different pretreatments and drying methods on the color of jujube powder. FD: vacuum freeze-drying; MPVD: microwave coupled with pulsed vacuum drying; HAD: hot air drying; CP: low temperature plasma pretreatment; US: ultrasonic pretreatment.

The color of jujube powder is one of the first sensory properties that consumers notice when purchasing. In Fig. 1. using the FD no pretreatment group as the control, it is observed that the samples dried by HAD no pretreatment group exhibits the highest color difference (ΔE) value of 15.31, followed by the CP + MPVD group, while the FD pretreatment group presents the lowest ΔE value, with no significant difference noted. The FD group has the highest L* value, whereas the L* value of microwave pulse vacuum dried samples is 7.6 % higher than that of HAD samples. This indicates that, compared to traditional drying methods, microwave pulse vacuum dried samples can maintain a brighter appearance. Additionally, the L* values of the HAD samples processed by CP and US increased by 10.2 % and 10.7 %, respectively, suggesting that pretreatment significantly enhances the L* value of jujube powder. This finding is consistent with the results reported by Sekhon et al. [28]. Regarding the a* value for MPVD samples, the order is no pretreatment < US pretreatment < CP pretreatment. This variation may be attributed to the heating effect during CP pretreatment, where the current applied to the samples surface raises the temperature, resulting in a higher a* value post-drying. The b* value, representing the yellow-blue spectrum, indicates that all samples appear yellow. The b* values across different drying methods are as follows: US pretreatment < CP pretreatment < no pretreatment. Notably, the b* value of the US + MPVD samples is superior to that of the samples without pretreatment, with a decrease of 2.24 %. Pretreatment showed a certain protective effect on color, which may be due to its reduced drying time and minimization of the Maillard reaction, thereby maintaining color stability. The study reported by Salehi et al. [29] supports these findings, stating that various pretreatments are able to significantly affect color stability by changing drying time and heat exposure, as observed in eggplant slices.

### 3.2 Particle size distribution

Table 1. Differences in particle size often indicate variations in fiber structure and the associated physical and chemical properties, thereby influencing product quality [30]. Table 1. The D_0.5_ values under three different drying methods were 149.25 μm (HAD), 126.3 μm (MPVD), and 48.25 μm (FD), respectively. This finding aligns with results reported by Baariuc et al. [31], showing that HAD leads to significant samples shrinkage, resulting in larger particle sizes in sorghum flour due to texture hardening. Following pretreatment, the D_0.5_ values for each drying method decreased significantly. For the MPVD samples, the pretreatments of CP and US reduced the D_0.5_ by 24.8 % and 23.6 %, respectively. This indicates that the particle size of jujube powder can be effectively reduced through CP or US pretreatment. Wang et al. [32] reported similar findings, noting that maintaining structural integrity by reducing resistance to moisture migration resulted in finer particle sizes in jujube flour.

**Table 1:** Particle size distribution of jujube powder under different pretreatment and drying methods.

Pretreatment	Drying Method	D_0.1_/μm	D_0.5_/μm	D_0.9_/μm	Span
Control	FD	9.39 ± 0.31^f^	48.25 ± 0.19^e^	163.3 ± 2.02^f^	3.18 ± 0.05^c^
	MPVD	19.97 ± 0.02^a^	126.3 ± 1.90^b^	352.5 ± 1.41^c^	2.67 ± 0.04^d^
	HAD	18.69 ± 0.40^b^	149.25 ± 3.74^a^	375.6 ± 6.39^b^	2.55 ± 0.32^d^
CP	FD	8.45 ± 0.24^g^	40.34 ± 0.27^f^	169.80 ± 3.25^f^	3.99 ± 0.04^b^
	MPVD	11.72 ± 0.81^e^	94.91 ± 0.22^d^	407.40 ± 1.55^a^	4.25 ± 0.15^ab^
	HAD	11.39 ± 0.07^e^	109.20 ± 3.67^c^	226.23 ± 5.36^e^	2.02 ± 0.09^e^
US	FD	6.35 ± 0.19^h^	22.07 ± 1.58^g^	104.63 ± 1.00^g^	4.46 ± 0.29^a^
	MPVD	14.69 ± 0.52^c^	96.44 ± 1.69^d^	402.63 ± 8.09^a^	4.02 ± 0.06^b^
	HAD	13.83 ± 0.15^d^	92.89 ± 1.09^d^	329.00 ± 6.15^d^	3.39 ± 0.09^c^

The lowercase letters in the same column represent significant differences (p < 0.05).

### 3.3 Bulk density analysis

**Fig. 2:**
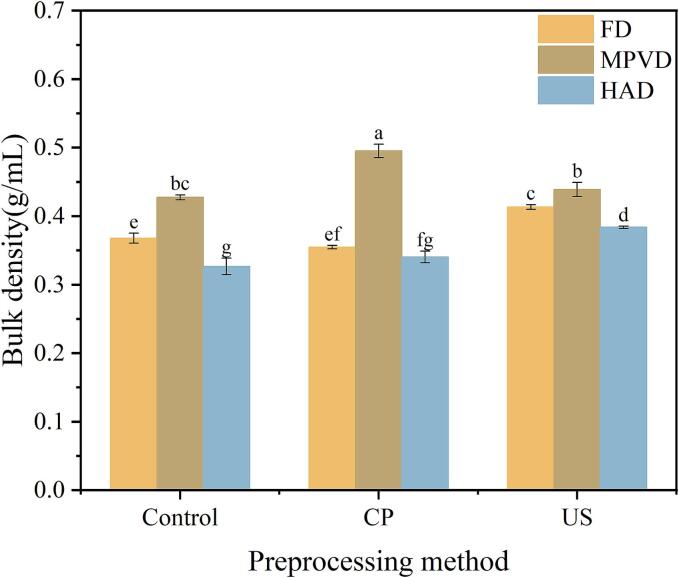

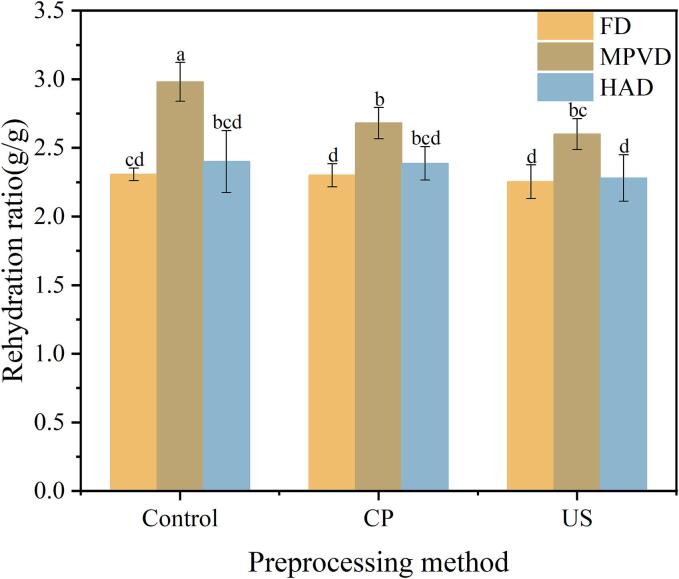

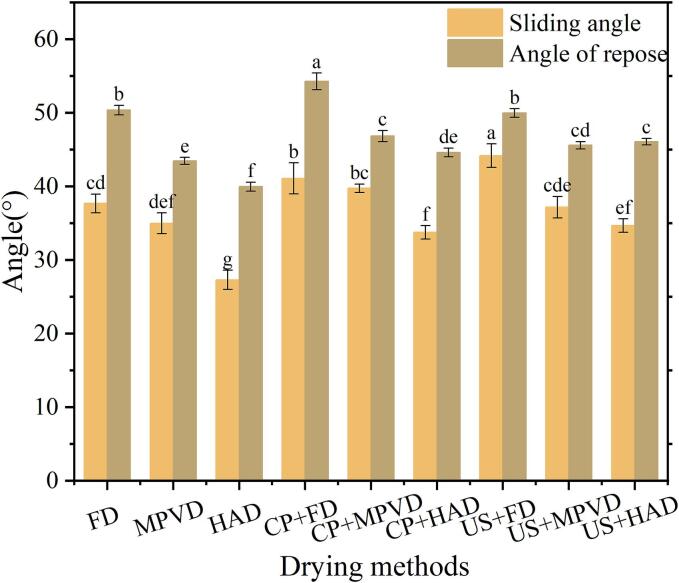

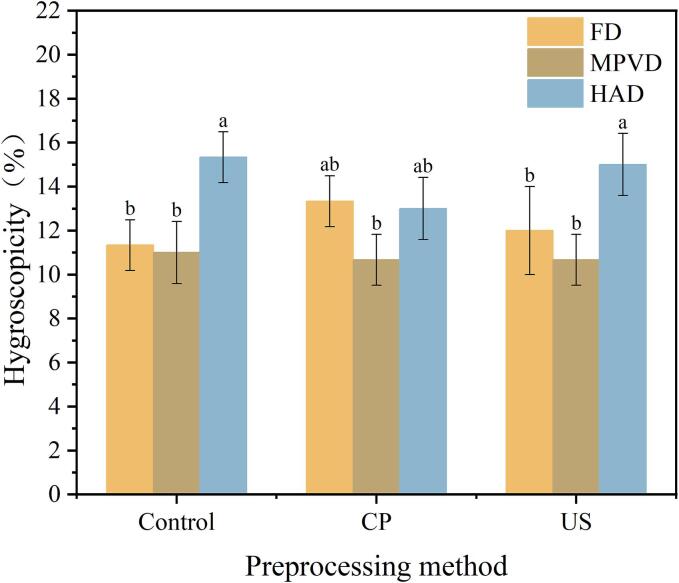
Effects of different drying methods on the physical properties of jujube powder; (a) represent the bulk density of jujube powder. (b) Represent the rehydration ratio of jujube powder. (c). Represent the flowability of jujube powder. (d) Represent the moisture absorption of jujube powder.

Bulk density plays a crucial role in the packaging and transportation of fruit and vegetable powders; higher bulk density typically reduces packaging and transportation costs. Fig. 2(a) shows the bulk density of jujube powder prepared using different pretreatment and drying methods. Among the various drying techniques, MPVD resulted in the highest bulk density (0.427 g/mL), followed by FD with a bulk density of 0.367 g/mL. HAD produced the lowest bulk density (0.326 g/mL) among the three methods. For MPVD and HAD samples, the application of pretreatments increased the bulk density of jujube powder compared to samples without pretreatment. Notably, the combination of cold plasma pretreatment with MPVD (CP + MPVD) resulted in the highest bulk density at 0.495 g/mL. Additionally, US + FD yielded a bulk density of 0.413 g/mL, which was higher than that of FD samples without pretreatment. These findings suggest that pretreatment can enhance the bulk density of jujube powder, potentially improving its suitability for commercial packaging and transport. Dev et al. [33] reported similar findings, highlighting that certain pretreatment techniques, followed by appropriate drying methods, can improve the structural integrity of powder particles, thereby increasing bulk density, as observed in spray-dried jujube powder. Similarly, Tajane et al. [34] noted that optimizing pretreatment and drying conditions is essential for achieving the desired bulk density in neem fruit powder, supporting the current study’s observation that bulk density can be effectively modulated by pretreatment and drying methods.

### 3.4 Rehydration ratio analysis

The rehydration ratio is an essential indicator of the quality of dried products, as it reflects the ability of the powder to absorb water and regain its original texture. Fig. 2(b) shows the impact of different pretreatment and drying methods on the rehydration ratio of jujube powder. Among the drying techniques, MPVD samples exhibited the highest rehydration ratio (2.98 g/g), followed by the HAD samples (2.4 g/g). In comparison, the FD jujube powder had a rehydration ratio of 2.31 g/g, indicating that the drying method significantly influences the rehydration capability of jujube powder. In terms of different pretreatments, both CP and US pretreatment methods significantly reduced the rehydration properties across the three drying methods. Specifically, FD samples exhibited reductions of 0.2 % and 2.3 %, HAD samples showed reductions of 0.5 % and 5 %, and MPVD samples experienced reductions of 10 % and 12.8 %. It is possible that the physical and chemical structure of the samples was compromised during the pretreatment process, leading to a decrease in rehydration. This finding aligns with results reported by Cui et al. [35].

### 3.5 Analysis of repose angle and sliding angle

The repose angle and sliding angle are critical parameters for evaluating the flowability of jujube powder, as shown in Fig. 2(c). According to Sun et al. [24], smaller repose and sliding angles indicate better flowability, with angles below 30° representing excellent flow characteristics and angles above 40° meeting the minimum requirements for industrial flow needs. In this study, the sliding angle was lowest for HAD samples at 27.29°, followed by MPVD at 34.98°, while FD jujube powder had the highest sliding angle at 37.68°. For samples dried by the same method but with different pretreatments, both FD and HAD samples exhibited the same trend in sliding angles. In MPVD samples, the sliding angle was smallest in the control group, followed by the US-pretreated group (37.17°), with the CP-pretreated jujube powder exhibiting the highest sliding angle (39.74°). The trend observed for repose angles across different drying methods was like that of sliding angles. Among HAD samples, the lowest repose angle was 39.97°, while FD samples with CP pretreatment had the highest repose angle at 54.26°. Yadav et al. [36] reported that finer particle sizes in dried okra powder resulted in larger repose and sliding angles due to increased particle adhesion, which limited flow. The current study confirms these findings, suggesting that optimizing particle size and drying method can significantly improve the flowability of jujube powder, particularly for applications requiring efficient handling and processing.

### 3.6 Water holding capacity (WHC) and solubility analysis

Table 2 shows the WHC and solubility of jujube powder under various pretreatments and drying methods, with FD and HAD used as reference points. Among the drying techniques, MPVD jujube powder exhibited the highest WHC at 2.8 g/g, followed by FD, with HAD samples showing the lowest WHC. In the FD group, the CP-pretreated jujube powder had the highest WHC at 2.58 g/g, followed closely by the US-pretreated group (2.44 g/g). Notably, the US + MPVD samples displayed the highest WHC across all samples at 2.93 g/g. The reduction in WHC observed in FD samples may result from the smaller particle size, which decreases the binding force between particles and water, a finding that aligns with research conducted by Hematian et al. [37], who reported similar effects in jujube powder after ultrasonic and microwave treatment. In terms of solubility, FD jujube powder exhibited the highest solubility at 89.33 %, followed by HAD and MPVD powders. The lower solubility of MPVD jujube powder compared to HAD samples may be attributed to its smaller particle size, which enhances the water-binding capacity of the powder and consequently increases WHC. However, this smaller particle size also strengthens interparticle interactions and promotes molecular aggregation, ultimately reducing water solubility. Pui et al. [38] similarly noted that a reduction in particle size and an increase in WHC in fruit powders, resulted in stronger cohesion between particles, thereby diminishing the ease with which the powder dissolves in water.

**Table 2:** Effects of different pretreatment and drying methods on the water holding capacity and solubility of jujube powder.

Pretreatment	Drying Method	Water holding capacity/ (g/g)	Solubility/ %
Control	FD	2.33 ± 0.02^c^	89.33 ± 2.31^a^
	MPVD	2.8 ± 0.13^a^	58.67 ± 2.31^f^
	HAD	2.13 ± 0.12^d^	72.00 ± 4.00^cd^
CP	FD	2.58 ± 0.02^b^	81.33 ± 2.31^b^
	MPVD	2.81 ± 0.061^a^	66 ± 2.82^de^
	HAD	2.29 ± 0.23^cd^	70 ± 2.82^d^
US	FD	2.44 ± 0.11^bc^	84.00 ± 4.00^ab^
	MPVD	2.93 ± 0.08^a^	61.33 ± 2.31^ef^
	HAD	2.4 ± 0.07^c^	78.00 ± 2.83^bc^

The lowercase letters in the same column represent significant differences (p < 0.05).

### 3.7 Hygroscopicity analysis

Hygroscopicity is a critical factor influencing the processing characteristics and storage stability of jujube powder. Higher hygroscopicity indicates a shorter storage period and a greater tendency to form clumps, which can adversely affect long-term storage and commercial viability. As shown in Fig. 2(d). The hygroscopicity of jujube powder varied significantly with different drying methods. HAD resulted in the highest hygroscopicity, with an average of 14.43 %, followed by FD at an average hygroscopicity of 12.2 %. The lowest hygroscopicity was observed in MPVD samples, ranging from 10.6 % to 11 %. Addo et al. [39] also reported that minimizing hygroscopicity is essential for reducing caking behavior in jujube powder, suggesting that drying methods preserving structural integrity, like MPVD, are more favorable for long-term stability. Within the same drying method, there were no significant differences in hygroscopicity across different pretreatment groups. However, among all samples, the highest hygroscopicity was observed in the HAD control group (no pretreatment) at 15.3 %, while MPVD samples with CP and US pretreatments exhibited the lowest hygroscopicity (10.67 %). This trend suggests that combining MPVD with pretreatments like cold plasma or ultrasound can further reduce hygroscopicity, enhancing the powder’s suitability for storage and reducing the likelihood of clumping. Pandey et al. [40] similarly found that optimized drying conditions reduce hygroscopicity in fruit powders, improving their shelf life and commercial usability.

### 3.8 Microstructure analysis

**Fig. 3:**
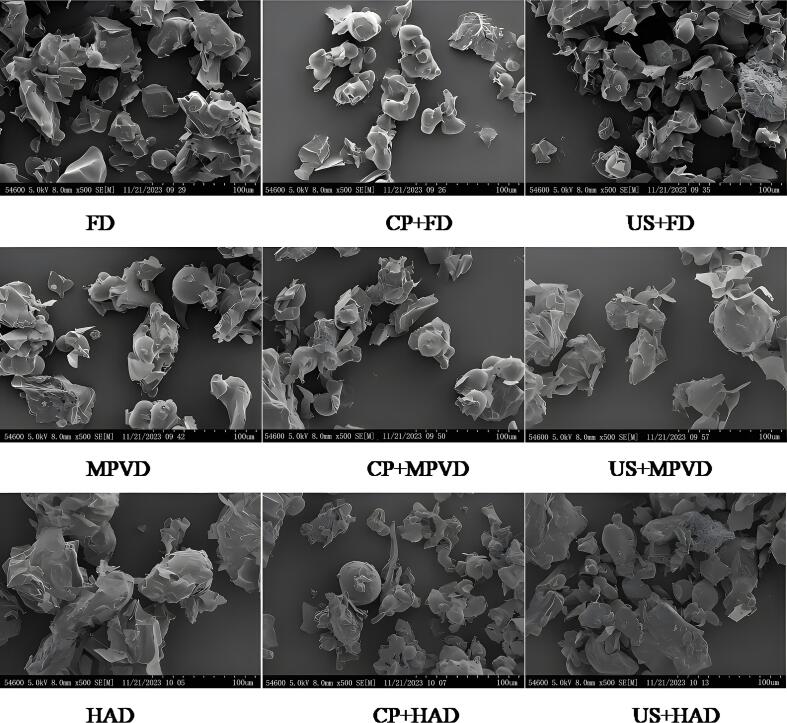
Microstructure of jujube powder under different pretreatment and drying methods Note: FD: vacuum freeze-drying; MPVD: microwave coupled with pulsed vacuum drying; HAD: hot air drying; CP: low temperature plasma pretreatment; US: ultrasonic pretreatment.

Fig. 3 displays the microstructures of jujube powder produced under different pretreatments and drying methods. in the had samples, a rough surface and the presence of rod-like particles with noticeable agglomeration were observed, likely due to the high drying temperature causing shrinkage and cohesion among particles. in contrast, the mpvd jujube powder displayed a more uniform particle distribution with smoother surfaces, and the particles were loosely arranged without signs of agglomeration. fd samples exhibited the smallest particle sizes, corresponding with the smallest particle size distribution observed in fd powder. Among the various pretreatment methods, CP + MPVD jujube powder appeared looser with smaller particles, potentially due to the reduced particle size and increased electrostatic repulsion between particles, which prevented clumping. For FD and HAD powders, the US pretreatment group showed smaller particles than the CP pretreatment group, suggesting that ultrasonic treatment may enhance particle fragmentation and dispersion. These findings are consistent with studies conducted by Moghbeli et al. [41], showing that spray-dried jujube powder produced with pectin-whey protein complexes resulted in a smoother and more uniform microstructure. Similarly, Seerangurayar et al. [5] found that foam-mat freeze-dried jujube powder using carrier agents led to better flowability and a more homogeneous microstructure, aligning with the smooth and non-agglomerated particles observed in MPVD and FD samples. These results highlight the importance of drying methods and pretreatment in achieving desirable microstructural properties in jujube powder for enhanced functionality in food applications.

### 3.9 Cation exchange capacity analysis

**Fig. 4:**
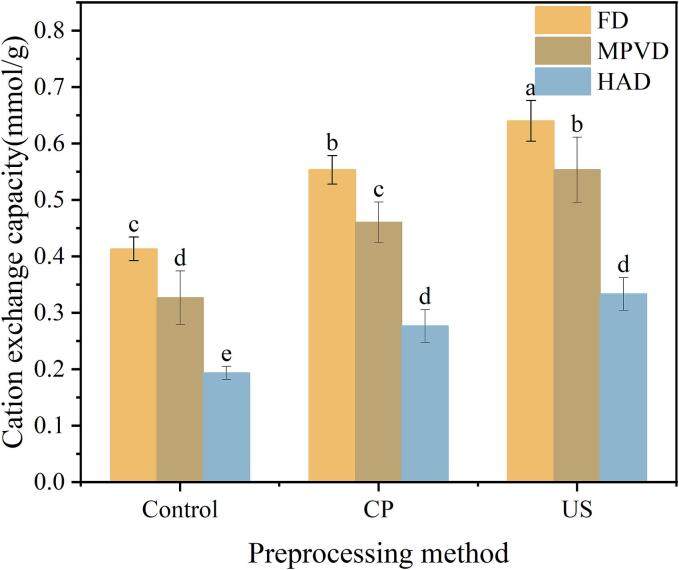
The effect of different pretreatment and drying methods on the cation exchange capacity of Jujube powder.

The cation exchange capacity (CEC) of jujube powder, influenced by different pretreatments and drying methods, is illustrated in Fig. 4. A higher CEC value indicates a greater nutritional potential of jujube powder. Among the drying methods, FD showed the highest CEC at 0.413 mmol/g, followed by MPVD at 0.326 mmol/g, with HAD resulting in the lowest CEC at 0.193 mmol/g. This may be due to the lower temperature environment in FD and MPVD, which minimizes the loss of bioactive compounds. Additionally, the vacuum conditions during freeze drying help preserve the porous honeycomb structure, while the negative pressure in MPVD maintains a similar structural integrity, allowing finer grinding and increased surface area for cation exchange.

Regarding pretreatment, in particular, MPVD samples with CP and US pretreatments showed a higher CEC (0.46 and 0.553 mmol/g, respectively) than the FD samples without pretreatment, indicating that pretreatment can further enhance the cation exchange capacity. This suggests that combining specific pretreatments with drying methods, such as MPVD, may yield jujube powder with enhanced nutritional and functional properties, making it a valuable addition to health-focused food products.

### 3.10 Analysis of total phenolic and flavonoid content

**Fig. 5:**
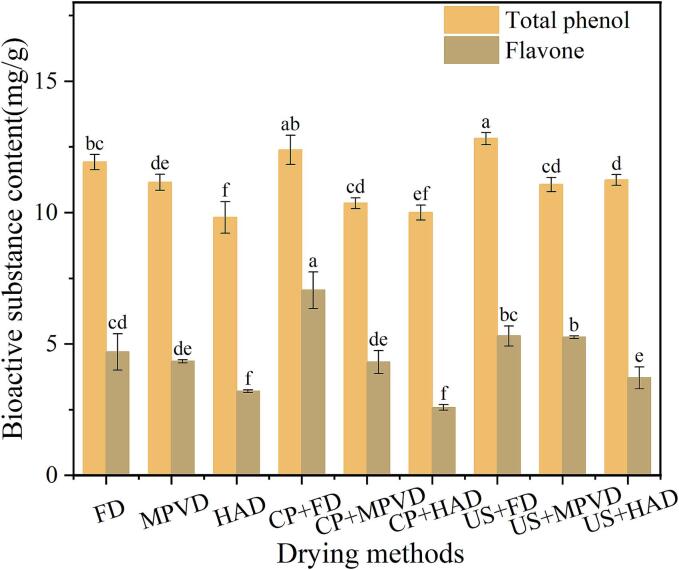
The effect of different pretreatment and drying methods on the total phenolic and flavonoid content in Jujube powder.

Flavonoids and phenolic compounds possess strong antioxidant activity. Measuring the content of flavonoids and phenolic compounds can directly reflect the level of antioxidant capacity, providing a basis for consumers to select foods with higher nutritional value. Fig. 5 shows the effects of different pretreatments and drying methods on the total phenolic and flavonoid content in jujube powder. Regarding drying methods, FD samples displayed the highest levels of total phenolic and flavonoid content, followed by MPVD, while HAD samples showed the lowest content. This difference may result from the high temperatures and extended processing times in HAD, which can lead to the deactivation of bioactive compounds and cause shrinkage of surface structures, thus impeding the release of phenolics and flavonoids.

Samples subjected to pretreatment exhibited higher total phenolic and flavonoid content compared to untreated samples. The highest flavonoid content was observed in the CP + FD samples, reaching 6.38 mg RE/g DW. In MPVD and HAD samples, US samples showed relatively high flavonoid content, with values of 5.21 and 3.40 mg RE/g DW, respectively. Among the pretreatments, US pretreatment led to the highest phenolic content, particularly in the US + FD samples, which reached 11.97 mg GAE/g DW. This may be attributed to the ultrasonic vibrations resonating with molecular structures within the material, creating microchannels that facilitate the release of bioactive compounds. Yang et al. [42] reported similar findings, demonstrating that ultrasonic pretreatment enhances the extraction of phenolics by disrupting cell structures. So, selecting appropriate pretreatments and drying methods to maximize the nutritional quality of jujube powder, particularly in terms of its bioactive content.

### 3.11 Flavor compound analysis

Table 3 presents the volatile compounds identified in jujube powder produced under various pretreatments and drying methods, revealing a total of 38 distinct flavor compounds, with alkanes and olefins as the primary constituents. In MPVD samples, the concentration of alkanes was notably high, accounting for 15.56 %–21.20 % of the total volatiles, surpassing that of FD samples (13.54 %–14.50 %) and significantly higher than HAD samples, which had the lowest alkane content. FD jujube powder exhibited relatively high levels of aldehydes and olefins, with aldehydes contributing to a characteristic grassy aroma unique to winter jujube. The lower aldehyde content in HAD samples may result from the oxidation of fatty acids and the metabolism of amino acids during the high-temperature drying process.

**Table 3:** Volatile compounds and relative contents of Jujube powder with different pretreatment and drying methods.

Number	Compound name			Analysis of volatile components and content of microwave pulsating vacuum dried winter Jujube powder under different pretreatments (%)																													
				FD			CP + FD			US + FD					MPVD			CP + MPVD					US + MPVD			HAD			CP + HAD				US + HAD
	**Taxanes**			**13.54**			**14.35**			**14.50**					**21.20**			**15.56**					**19.17**			**13.08**			**13.97**				**12.19**
1	Hexamethylcyclotriosiloxane			6.04			5.57			6.43					6.50			7.86					6.46			7.31			6.88				7.60
2	Octamethylcyclotetrasiloxane			2.11			−			−					0.63			−					−			−			−				−
3	Cyclotetrameric dimethyl siloxane			−			−			−					−			1.47					1.26			0.77			0.70				1.38
4	Undecane			1.59			−			−					3.87			−								2.36			2.02				0.81
5	Cyclopentadimethylsiloxane			−			−			−					1.18			1.08					0.70			0.56			0.46				0.57
6	1,3-*trans*-dimethylcyclopentane			−			−			−					0.75			−					−			−			−				−
7	N-dodecane			1.88			2.76			1.39					2.73			1.40					1.27			1.08			1.31				1.20
8	Tetradecane			−			0.45			−					−			−					0.53			0.51			0.40				−
9	N-hexadecane			0.40			−			−					−			0.48					−			−			0.43				−
10	Tetradecyl cycloheptasiloxane			−			1.13			1.73					1.42			1.10					0.97			−			1.26				−
11	Dodecyl cyclohexyl siloxane			0.61			3.47			4.40					2.42			1.65					1.76			0.49			0.51				0.63
12	Tridecane			0.90			0.97			0.54					1.08			0.51					−			−			−				−
13	Ethyl alkane			−			−			−					−			−					6.22			−			−				−
14	N-nonadecane			−			−			−					0.61			−					−			−			−				−

	**Aldehyde**			**9.45**			**11.85**			**11.39**					**1.33**			**1.54**					**1.06**			**0.93**			**1.05**				**1.67**
1	Trans-2-vinylaldehyde			7.39			8.35			9.15					1.33			1.54					−			0.93			−				0.82
2	Benzaldehyde			1.24			1.29			1.22					−			−					1.06			−			1.05				0.85
3	N-hexanal			−			0.88			−					−			−					−			−			−				−
4	Decanal			0.82			1.32			1.02					−			−					−			−			−				−

	**Alkenes**			**9.55**			**16.88**			**9.53**					**8.13**			**8.65**					**6.92**			**3.04**			**4.29**				**3.33**
1	Benzo cyclobutene			−			−			9.53					−			−					−			−			−				−
2	Styrene		9.55			7.86					−				−		4.69					3.37			−			−				−	
3	(+) − Limonene			−				9.02				−		8.13					3.96		3.55					3.04				4.29			3.33

	**Aromatic hydrocarbons**			**13.95**				**20.78**				**20.76**		**25.86**					**27.03**		**29.84**					**32.73**				**23.82**			**34.97**
1		O-xylene			5.98				2.32				5.07			−				7.26				−			19.58				6.62		−
2		Ethylbenzene			1.27				−				4.51			4.59				4.69				−			5.59				4.00		5.73
3		Meta xylene			6.70				18.46				5.20			21.27				6.64				13.36			−				5.42		17.33
4		P-xylene			−				−				5.98			−				8.43				16.48			7.56				7.79		11.91

		**Esters**			**9.10**				**3.24**				**3.70**			**1.80**				**1.66**				**1.94**			**1.86**				**1.81**		**1.79**
1		Ethyl octanoate			2.43				1.82				1.86			1.80				1.66				1.94			1.86				1.81		1.79
2		Diethyl succinate			0.81				1.42				−			−				−				−			−				−		−
3		Ethyl decanoate			1.03				−				1.29			−				−				−			−				−		−
4		Ethyl undecylate			−				−				0.56			−				−				−			−				−		−
5		Dibutyl phthalate			3.68				−				−			−				−				−			−				−		−
6		Ethyl palmitate			1.15				−				−			−				−				−			−				−		−

		**Alcohols**			**1.62**				**0.78**				**0.71**			**0.00**				**0.82**				**0.86**			**0.77**				**0.78**		**0.78**
1		DL menthol			0.63				0.78				0.71							0.82				0.86			0.77				0.78		0.78
2		Phenylethano			0.98				−				−			−				−				−			−				−		−

		**Acids**			**9.90**				**5.90**				**2.29**			**0.00**				**5.57**				**4.29**			**2.44**				**1.77**		**1.81**
1		Hexanoic acid			3.51				3.36				2.29			−				5.57				4.29			2.44				1.77		1.81
2		bitter			4.94				2.55				−			−				−				−			−				−		−
3		N-decanoic acid			1.45				−				−			−				−				−			−				−		−

		**Other categories**			**0.64**				**−**				**0.82**			**0.00**				**1.36**				**0.68**			**4.97**				**1.05**		**1.60**
1		Methyl heptene ketone			−				−				0.82			−				−				0.68			0.69				−		0.89
2		Beta Dupine			−				−				−			−				1.36				−			4.28				1.05		0.70

FD: vacuum freeze-drying; MPVD: microwave coupled with pulsed vacuum drying; HAD: hot air drying; CP: low temperature plasma pretreatment; US: ultrasonic pretreatment- indicates not detected.

Aromatic hydrocarbons were most abundant in HAD samples, followed by MPVD samples. HAD and CP + MPVD samples contained distinctive beta-cadinene, a terpene compound that imparts an herbal aroma to jujube powder. FD samples, on the other hand, had the highest concentration of DL-menthol, followed by CP + MPVD samples, giving the powder a minty, woody fragrance. The increased content of volatile compounds in CP-pretreated samples across all drying methods suggests that CP pretreatment may enhance the retention of aroma compounds by facilitating the release of volatile constituents from cellular structures. Additionally, five aromatic hydrocarbons were identified in CP + MPVD, HAD, and US + FD samples, representing 27.03 %, 34.97 %, and 20.76 % of the major compounds, respectively. This finding aligns with research studied by Wang et al. [43], which demonstrated that MPVD can effectively preserve the quality and unique aroma of jujube by retaining specific volatile compounds. Afifah et al. [44] also reported that pretreatment methods prior to drying improve the preservation of flavor compounds in dehydrated foods, such as ground beef, by minimizing the loss of volatile constituents.

These results underscore the importance of pretreatment and drying methods in influencing the volatile profile of jujube powder. While MPVD and CP pretreatment notably enhance the retention of alkanes and unique terpenes, FD preserves aldehydes and menthol, contributing to a distinct aromatic profile in jujube powder. This comprehensive understanding of volatile compound retention is essential for optimizing the sensory quality of jujube powder in various applications.

## 4 Conclusion

In terms of physical properties, MPVD jujube slices outperformed FD slices in water-holding capacity, flowability, rehydration capacity, and bulk density. Regarding chemical properties, the US-FD jujube slices had the highest cation exchange capacity and total phenolic content, while the CP-MPVD jujube slices contained the highest flavonoid content. Additionally, MPVD jujube slices pretreated with CP and US exhibited higher cation exchange capacities compared to the untreated FD slices, suggesting that CP and US pretreatments could enhance the cation exchange capacity and phenolic and flavonoid contents. Analysis of volatile compounds revealed significant differences among the drying methods and pretreatments. FD slices retained higher levels of aldehydes and olefins, MPVD slices had a greater proportion of alkanes, and HAD slices contained the highest number of aromatic hydrocarbons) particularly beta-cadinene with its characteristic woody aroma. Among the pretreatments, jujube slices pretreated with CP showed better retention of flavor compounds.

In conclusion, the appropriate selection of pretreatment and drying methods can remarkably enhance the quality and functional properties of jujube slices. The combination of MPVD with CP or US holds great promise for manufacturing high-quality jujube slices with enhanced nutritional and sensory qualities. This indicates that they can be valuable tools in the food industry to develop jujube-based products with enhanced functional and sensory attributes, meeting the growing consumer demand for nutritious and delicious food products. Therefore, further research and development in this area are highly recommended to fully explore the potential of these pretreatment and drying methods for the large-scale production of high-quality jujube slices.

## CRediT authorship contribution statement

**Zhengdong Wan:** Writing – review & editing, Writing – original draft. **Zhuofan Ji:** Writing – original draft. **Dandan Zhao:** Writing – review & editing, Writing – original draft, Supervision. **Yamei Liu:** Supervision. **Zhentao Zhang:** Supervision. **Jianxiong Hao:** Supervision.

## Declaration of competing interest

The authors declare that they have no known competing financial interests or personal relationships that could have appeared to influence the work reported in this paper.

## Data Availability

The data used and analyzed in this study can be obtained upon reasonable request from the corresponding author. Please contact Wan Zhengdong at 1504023063@qq.com to obtain the data.
